# Iron mishandling in the brain and periphery in Parkinson’s disease

**DOI:** 10.1038/s41531-025-01089-7

**Published:** 2025-08-18

**Authors:** MacKenzie L. Bolen, Kelly B. Menees, Aidan C. Dupreez, Malú Gámez Tansey

**Affiliations:** 1 Center for Translational Research in Neurodegenerative Disease, College of Medicine, University of Florida, Gainesville, FL USA; 2 Department of Neuroscience, College of Medicine, University of Florida, Gainesville, FL USA; 3 McKnight Brain Institute, University of Florida, Gainesville, FL USA; 4 Aligning Science Across Parkinson’s (ASAP) Collaborative Research Network, Chevy Chase, MD 20815 USA; 5 Department of Neurology, Indiana University School of Medicine, Indianapolis, IN USA; 6 Stark Neuroscience Research Institute, Indianapolis, IN USA; 7 Norman Fixel Institute for Neurological Diseases, University of Florida, Gainesville, FL USA

**Keywords:** Immunology, Neuroscience, Pathogenesis, Risk factors, Signs and symptoms

## Abstract

The heterogeneous prodromal phase of Parkinson’s disease (PD) has made identifying catalysts that drive disease progression critical for effective development of disease-modifying therapies. Recently, the role of gastrointestinal dysfunction in inflammation that drives neurodegenerative risk has gained attention as a target for intervention. However, to date, there have been no clear internal or environmental catalysts identified in the gut that drive risk for PD. Here, we review the literature on iron dysregulation in the brain, blood, and gut in PD and propose that iron dysregulation outside the brain is an important catalyst that may represent a prodromal mechanistic link in gut-first PD.

## Introduction

Parkinson’s disease (PD) is commonly characterized by the loss of dopaminergic (DA) neurons in the substantia nigra (SN)^1^, which leads to the canonical motor symptoms (i.e., bradykinesia, tremor and rigidity) associated with this devastating disease^2^. Most PD cases are idiopathic^3^, suggesting a highly complex multifaceted etiology. Due to the complex network of PD risk factors, several groups have looked outside the brain for early nonmotor signs of PD^4,5^. Efforts in the search for early biomarkers of PD prior to the onset of DA neuronal loss have resulted in a large breadth of literature characterizing PD as a multi-system disorder^6^. Increasing evidence bolsters the critical role of chronic systemic inflammation (CSI) in the pathogenesis of PD^7^. One such inflammatory risk factor of PD is gastrointestinal (GI) dysfunction, which can occur up to 20 years prior to PD diagnosis and in up to 80% of individuals living with PD^8^. Moreover, it has recently been suggested that for some individuals, PD may start in the gut, termed “body-first” PD^9^. There is still no early identifier or fully validated mechanism that explains why only a subset of individuals living with PD develop GI dysfunction prior to onset of motor symptoms and whether arresting the pathology in the prodromal stages can prevent progression to motor deficits.

### Iron handling in the brain of those with PD

Iron is critical for neuronal function and is transported from the periphery into the brain via endothelial cell absorption along the vasculature through iron transport proteins (such as transferrin)^10^. Pathologic iron deposits in the substantia nigra (SN) of post-mortem tissue from PD patients were discovered prior to the formation of the canonical dopamine (DA) hypothesis^11^. Specifically, it is known that DA neurons within the SN have also been shown to have excess iron in post-mortem SN tissue from PD patients via electron probe x-ray microanalysis^12^. Iron is directly involved in the regulation of tyrosine hydroxylase (TH) to dihydroxyphenylalanine (DOPA) and eventually DA, thus, disturbances in iron concentrations within the brain can directly impact DA production in the brain. Also, an increase of iron specifically in the SNpc leads to increased lipid peroxidation, free radical formation, and neuronal death—all of which contribute to neurodegenerative processes^13^. Additional previous work has linked the accumulation of ferric iron to pathologic aggregation of several neurodegenerative-associated proteins, such as *α*-synuclein (α-syn), tau^14^, amyloid beta (Aβ)^15^, and TDP-43^16^ in *post mortem* tissue and diagnostic imaging studies. Additionally, excess iron housed in the SN has also been shown to correlate with the formation of Lewy bodies in post-mortem tissue from PD patients^17^. Specifically, excess free iron can oxidize α-syn and cause the oligomer to aggregate, leading to intracellular inclusions or Lewy bodies^18,19^. Aggregated α-syn can drive ferroptosis (iron-mediated cell damage and eventual death) by disrupting lysosomal trafficking and preventing the removal of excess iron from the brain^20,21^. Ferroptosis is an independent cell death pathway, where iron-dependent lipid peroxidation results in oxidative stress, of which DA neurons are highly sensitive to ref. ^22^. Brain regions associated with motor function, such as the basal ganglia, tend to have higher iron content than nonmotor-associated regions^23^. Diagnostic imaging, such as MRI or PET, has been influential in identifying a distinct increase in iron deposition within the SN of those living with PD^24^. Using transcranial ultrasound and evaluating hyper-echogenicity as a surrogate for excess iron, multiple studies have found a significant increase in iron deposits in the SN of those living with PD as compared to healthy controls^25,26^. Additionally, specialized MRI-T2 or SWI and TSPO-PET imaging has been implemented to measure brain microglial activation as a surrogate for inflammation to identify prodromal phases of PD^27–29^. Of which, excess iron accumulation within microglia is known to polarize microglial activation to a pro-inflammatory phenotype^30^. In vivo imaging of both iron and neuroinflammatory activity seems to be the next step in brain region-specific identification of the role these biomarkers play in the progression of PD.

Excess ferric iron is housed in ferritin, which is the dominant ferric iron storage protein and is thought of as a surrogate for total iron content^31^. Moreover, it is known that activated immune cells are critical in neuroinflammation. Interestingly, these same infiltrating immune cells have been reported to have excess ferritin in the brain^32,33^. Several reports have identified individuals with hereditary peripheral iron overload, hemochromatosis, have an increased risk for idiopathic PD^34,35^; indicating a direct link between iron metabolism and PD etiopathogenesis. There is discordant literature regarding ferritin load in the cerebrospinal fluid (CSF) of those living with PD. One study identified a correlational decrease in CSF ferritin load and an increase in iron deposition in the SN of those living with PD (as compared to control)^36^. A separate study also found ferritin concentration increased in CSF with progression of motor symptom severity^37^, indicating a role of dysregulated iron accumulation in those living with PD. It is known that PD develops over decades, the different outcomes of these studies could be difference in stage of disease progression or even average age of the patients enrolled in the study. Future studies that enable a thorough longitudinal assessment of how both peripheral and brain iron change over time in the context of disease will be critical if iron is to be used as a peripheral biomarker of PD risk or disease progression.

There appears to be a synergy between toxic iron accumulation and inappropriate immune cell activation, reactive oxygen species (ROS) production and lipid peroxidation represented by iron handling at the level of the brain and blood in those living with PD^38,39^. A working hypothesis stipulates that this iron accumulation is due to trafficking breakdown of genes and proteins associated with immune activation early in PD pathogenesis, resulting in peripheral pro-inflammatory priming of the brain^40,41^.

### Iron handling in peripheral circulation and immune cells from those living with PD

Investigating biosamples from outside of the brain prior to the onset of PD-related neuropathology will help characterize disease progression and aid in therapeutic investigation. Following chronic antigen exposure, immune cells often display phenotypes that can be used to stage disease or predict future trajectories^42–45^. We posit that these phenotypes could inform about or herald future disease in or progression into the brain, such that immune cells in those living with PD may display critical transcriptional, population abundance, and inflammatory profiles similar or with some overlap as those observed in *post mortem* brains of individuals with PD or their CSF samples^7^. A wealth of literature now supports the idea that these easily accessible samples could be leveraged to investigate features of the immune cell inflammatory milieu as early biomarkers of disease risk prior to PD based on ample evidence that 1) PD is a disease often characterized by multi-organ involvement and inflammation evinced in the peripheral immune system, and 2) these inflammatory and immune dysregulation indicators can be detected far before the onset of frank neurodegeneration.

Several studies have identified excess total iron in the brain but decreased transferrin, total iron, and ferritin in serum^46–48^ and plasma^49^ of those living with idiopathic PD. The authors identified no difference in circulating iron as a function of dietary intake. This discrepancy is likely due to dyshomeostasis in peripheral iron metabolism and likely expedites iron entry into the brain^36^. Hair samples from those living with PD also display decreased iron, and these iron levels did not vary across age or disease duration^50^. Additionally, total iron from plasma samples, obtained from an additional cohort of individuals living with PD, revealed a significantly higher level of total iron content as compared to age- and sex-matched healthy controls. However, two other datasets revealed no difference in serum iron or ferritin levels between those living with idiopathic PD and healthy controls^51,52^. The difference in serum ferritin content as a function of PD pathogenesis may be due to the difference in cohort average disease duration or age. This emphasizes the need to continue to dissect a mechanism identifying the impact of iron mishandling in the periphery and the resulting effects on the neurodegenerative cascade known to occur in PD.

**Fig. 1:**
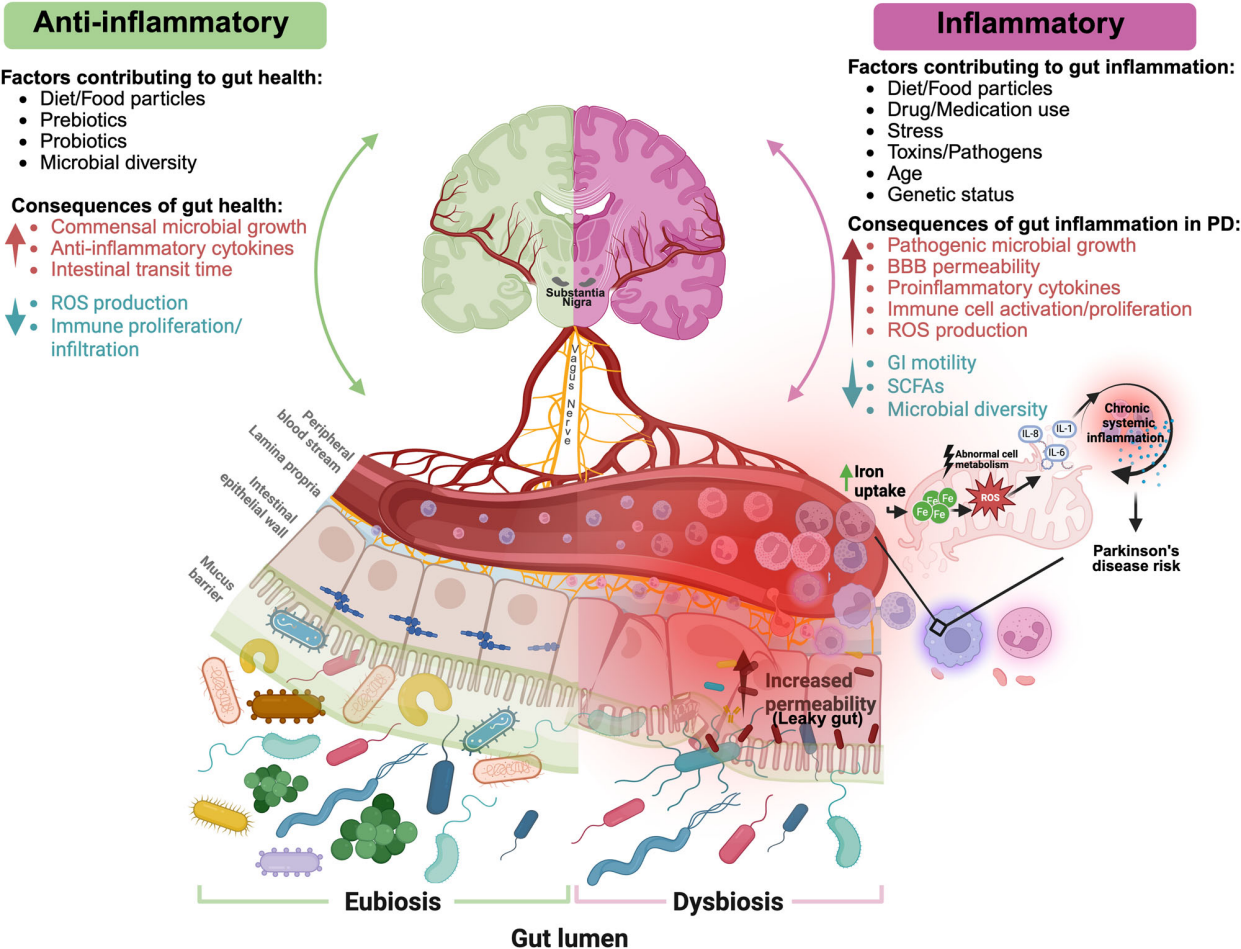
Proposed model of gut-blood-brain interface in Parkinson’s disease pathophysiology. In individuals with gastrointestinal (GI) dysfunction, decreases in microbial diversity often drive a decrease in commensal and an increase in transition to pathogenic microbiota. This dysbiosis can act as an inflammatory trigger that breaks down the intestinal epithelial wall and results in a leaky gut. Increasing pathogenic infiltrates drive aberrant immune-microbe interactions, generating a heightened immune response. Additionally, in the context of chronic remittent GI dysfunction, anemia of chronic disease (ACD) often occurs. ACD induces iron sequestration in mucosal immune cells. Iron overload drives reactive oxygen species (ROS) production and results in altered mitochondrial respiration. Mitochondrial stress increases cytokine release and further perpetuates chronic systemic inflammation. A chronically activated and dysregulated peripheral immune system presents a challenge to the brain due to disrupted central-peripheral neuroimmune crosstalk, which we hypothesize contributes to Parkinson’s disease (PD) etiopathogenesis. BBB blood brain barrier, GI gastrointestinal, SCFAs short chain fatty acids. This schematic was created using Biorender.com.

Immune cells act as the primary defense against pathogen infiltration through the epithelial gut lining, proliferating and dynamically responding as the innate security guards of the body. Increased intracellular iron accumulation becomes toxic in both enterocytes and gut mucosal resident immune cells (such as glia and monocytes)^53^. It is not yet known what the catalyst is for iron dysregulation in the periphery. One hypothesis is chronic immune stimulation, and consequential activation may be the driver, which is displayed in Fig. 1. Iron can play a critical role in inappropriately activating immune cells via^47^ 1) disruption in commensal bacteria concentration in the gut, 2) driving bioenergetic changes by supercharging mitochondrial electron transport chain (ETC) stress, and 3) driving cell death pathways such as ferroptosis^47^. Moreover, several reports suggest that ferroptosis induces the activation of the innate immune system^54^, driving a positive feedback loop of proinflammatory signaling. A primary location of this aberrant chronic stimulation is the gut barrier, the only site of iron absorption in the body. The endotoxin hypothesis of PD directly emphasizes this idea, where common toxicants such as pesticides, chemicals, or heavy metal exposure contained within processed foods are proposed to catalyze gut inflammation, resulting in immune system activation, and PD-associated neurodegenerative risk^55^ (Fig. 1).

### Iron handling in the gut of individuals living with PD

PD is a slowly progressing and complex disease known for its prolonged prodromal phase and complex etiopathogenesis^56^. Both excess iron deposition in the brain and immune dysregulation are well characterized brain phenotypes of PD; however, there are still no validated early biomarkers of PD risk or progression. One such source of CSI is at level of the gut, which leads to the hypothesis that a subset of PD cases may begin in the gut and eventual prevention or cures for these individuals should begin with a gut-first approach. We posit that biomarkers of iron handling, such as total iron^57^, hepcidin, transferrin, ferritin and haptaglobin^58^ could be indicators of neurodegenerative disease risk and should be evaluated in chronic systemic inflammatory diseases, like IBD.

The gut mucosa acts as a physical barrier to harmful pathogens^59^; however, in the context of dysbiosis, the integrity of the cell-cell epithelial junctions of the gut can become critically impaired^60^. The breakdown of the epithelial barrier that protects the host from infection allows for increased permeability of harmful antigens^61^ and is known as a “leaky gut”. This “leaky gut” catalyzes a proinflammatory immune response by increasing mucosal immune cell engagement with antigens present in the gut as well as allowing for gut microbial products to leak into the peritoneal cavity and the bloodstream^62^ (Fig. 1). GI dysfunction is an understudied and promising early-risk factor for a subset of those living with PD^63–69^ Constipation is the most common GI symptom associated with PD and can occur 15–24 years prior to the onset of motor features^70,71^. DA is a critical enteric neuromodulator that drives gastric motility^72^. Some literature supports a lack of peripheral enteric DA as a possible initiator for this early PD-associated constipation^73^. One study identified that middle-aged males who had less than one bowel movement a day had a four-fold increased risk for PD development over a 24 year period, as compared to age- and sex-matched counterparts with regular regimens^74^. Additionally, several studies have identified an increase in intestinal wall permeability in individuals with PD, as compared to age- and sex-matched controls^75,76^. However, this increased permeability was in the absence of gross intestinal morphological changes or damage, indicating the possibility for low-grade but chronic levels of peripheral inflammation in these individuals. To summarize these findings, the gut-first hypothesis of PD includes^7,77^ (Fig. 1): 1) an inflammatory trigger that induces GI dysbiosis and a leaky gut; 2) activation of the peripheral innate and adaptive immune systems; 3) increased infiltration of peripheral immune cells into the brain; 4) CSI-induced neuroinflammation via crosstalk with infiltrating immune cells and proinflammatory cytokines; and 5) neuroinflammation as a catalyst for PD onset in selectively vulnerable brain regions. Gut-associated proinflammatory triggers in human studies that have been correlated with PD risk include pesticide or toxic pollutant exposure^78^ as well as infection^79^. However, inflammatory bowel disease (IBD) is the most common GI-associated inflammatory condition that increases risk for PD, especially in the United States^80^. IBD is a disease of chronic relapsing inflammation within the intestinal mucosa. Remittent inflammation from IBD leads to breakdown of the epithelial intestinal lining and excessive release of both apoptotic and necrotic cell factors (i.e., chemokines and cytokines)^81^. Therefore, it is likely that the CSI induced by IBD is the basis for the epidemiological association between IBD and an increase in PD risk; where the immune system is proposed to be the communicating inflammatory messenger between the inflamed leaky gut, peripheral nervous system, and CNS^82^. In parallel to the rise in PD diagnoses, IBD diagnoses have increased 46% from 2006 to 2021—with the primary justification being change in diet composition^83^. However, other factors that trigger risk for PD along this gut-blood-brain axis are still unknown. Below, we will discuss iron mishandling due to GI dysfunction as another possible and important catalyst linking early GI dysfunction to PD pathogenesis (Fig. 1).

Interestingly, global iron homeostasis is regulated at the level of the GI tract, where tissue inflammation, blood loss, and an increase in inflammatory cytokines leads to 79–100% of individuals living with IBD developing anemia at least once during the course of their disease^84^. Furthermore, anemia is the most frequent metabolic complication of IBD^85^. Iron trafficking extracellularly is regulated by cytokine interaction; therefore, since IBD induces repeated phases of cytokine storms, IBD can lead to critical dysregulation of iron import and export receptors^86^. Inflammation-induced anemia or ACD, a type of chronic anemia induced by inflammation-associated disease, drives a cytotoxic increase in intracellular iron^87^. A possible inflammatory catalyst for iron loading in the periphery could be from ACD, which impairs complex I and II in the electron transport chain (ETC) within immune cells, induces excess ROS production^88^, increases immune cell activation, and proliferation^89^.

Microbial diversity and composition are tightly linked to host nutrient absorption, with a primary nutrient being iron^87^. Iron is a natural source of nutrition for bacteria; therefore, iron mishandling in the gut can and does lead to substantial dysbiosis^90^ (Fig. 1). However, as a positive feedback loop, dysbiosis of the gut microbiome has been correlated with an increase in susceptibility to ferroptosis via alteration in production of metabolites that modulate oxidative stress and overall dysregulation of systemic iron homeostasis^91^. For example, opportunistic pathogens or bacterium that can become pathogenic in dysbiotic conditions, consume more iron than commensal bacterial communitues in a eubiotic environment^91^.

A dysbiotic microbiome promotes ferroptosis^92^. The authors direct the reader to an in-depth review detailing the impact of a specific bacterium on ferroptosis^91^. A more pathogenic gut flora induces a more activated immune response by releasing a new complement of metabolites at the gut mucosal interfase^93^, connecting the increased immune activation seen in individuals with PD. One such example of metabolites associated with immune regulation that are depleted in the microbiome of those living with PD is short-chain fatty acids (SCFAs). SCFAs regulate intestinal permeability and thus regulate host intestinal immunity^93^. SCFAs also regulate iron solubility in the intestinal lumen by reducing intestinal pH. Thus, the depletion in SCFAs, already known to be associated with a diseased gut, likely also propagates a decrease in iron absorption^94^. Along the same lines, in an in vitro experiment testing adolescent stool samples depleted in iron, a decrease in *Roseburia*, *Clostridium Cluster IV*, *Bacteroides* and an increase in *Lactobacillus* and *Enterobacteriaceae* were reported, recapitulating a phenotype very similar to that seen in stool samples from those living with PD^95^. The findings from these studies are in conflict with two other studies that have reported low iron environments decreasing intestinal infection and catalyzing a more favorable environment for the growth of commensal or probiotic bacterium^58,96^. These discordant results are likely due to differences in methodologies, including differences in the ages of the populations recruited for the studies. Of importance is the lack of longitudinal analysis of peripheral iron handling in inflamed patients. To develop therapeutic strategies to prevent or reverse dysbiosis and mitigate risk for PD, there is a critical need to parse and define what a “healthy” microbiome looks like in a middle-aged population and how it changes as a function of aging.

The relationship between iron metabolism and the gut microbiome is likely to be a positive feedback loop, where dysbiosis can further generates excess cytosolic iron. One such example is an increase in production of the metabolite hydrogen sulfide (H_2_S) due to an overgrowth of pathogenic bacteria in the gut. Excess H_2_S aids in the breakdown of the mucosal lining in the gut and hyperpermeability of inflammagens^97^. H_2_S drives the release of cytochrome C oxidase from mitochondria, which increases free iron in the cytosol and induces ROS production (such as hydrogen peroxide)^98,99^. It is known that individuals living with PD have a generally less diverse and more pathogenic composition of gut flora^100^; iron mishandling likely plays a critical role in this dysbiosis.

Iron is a tightly regulated micronutrient that is fundamental to life-sustaining biochemical processes, such as mitochondrial function^101,102^. Specifically, iron-sulfur clusters can accept and transfer electrons along the ETC (specifically within complex I) in mitochondria, playing a crucial role in energy production^103^. Distinct mitochondrial morphology associated with iron mishandling includes: smaller mitochondria, increased mitochondrial membrane density, and decrease in mitochondrial cristae^104^. It is well known that PD is associated with mitochondrial complex I deficiency^105–107^, therefore iron mishandling in the periphery may catalyze the mitochondrial stress commonly observed in those living with PD.

### Gut-first approaches to therapy

All existing PD therapies only act to quell or slow symptoms from unrecoverable motor circuitry damage; there is no existing medication that halts or slows the progression of PD. Even so, the most used PD medication to replace the lost DA, levodopa, only supplies initial symptomatic relief, as over time the nigrostriatal circuit becomes resistant to excess DA relief. Gut dysfunction may be an early feature of “asymptomatic” PD as a non-motor phenotype present prior to the death of DA neurons; therefore, biomarker investigation and eventual therapeutic discovery are critical within this early prodromal stage of PD pathobiology. Iron chelators are actively being investigated as possible therapeutics for PD due to their ability to entrap, deactivate and export excess iron from the brain^108^. A current clinical trial using the iron chelator Deferiprone (FAIRPARK-II: NCT01539837)^109^ yielded no clinical benefit in individuals living with PD and in phase II, patients reported a worsening of motor symptoms as measured by Unified Parkinson’s Disease Rating Scale (UPDRS) scores. A recent update on the Deferiprone trial found that those living with PD who were taking levodopa/carbidopa while on Deferiprone had no change in motor symptoms; however, those not levodopa/carbidopa had a significant worsening of motor symptoms when taking Deferiprone^110^. Although iron chelators appear to delay disease progression in PD animal models^108,111^, there has been limited success in human trials. This may be due to the fact that DA-producing neurons require just the right amount of iron for cell metabolism^112^. Further investigation is warranted to understand the relationship between chelation and iron availability in both the brain and the periphery. Existing iron chelator studies have focused on a brain-first approach; however, we propose that for improved efficacy, these therapeutics should be targeted to iron mishandling in the periphery, prior to the unrecoverable loss of DA neurons in the brain. It should be noted, however, that there is no such thing as a truly metal-specific chelator, and these compounds affect a variety of different biologically relevant metals (i.e., Zn, Cu, Fe, Mn) with varying affinities. All these metals have important physiological roles, and their biology is tightly regulated; interfering with that regulation could lead to unanticipated secondary effects. Fig. 1.

One potential gut-targeted treatment option is the assessment of patient diet coupled with existing PD therapies (including DA replacement therapy or surgical interventions like deep brain stimulation) to provide a more efficacious outcome. A diet aimed at restoring commensal bacterial colonies to minimize development of leaky gut, and that can provide nutrients to produce anti-inflammatory metabolites (such as SCFAs) should be considered. One example is a diet high in fiber and fermented foods, such as forms of the mediterranean diet. A retrospective study evaluating those consuming a mediterranean or mediterranean-like diet found an inverse correlation between prodromal PD features in those on a healthier diet, as compared to a classic western diet^113^. Direct investigation of the mediterranean diet and PD onset and/or progression is needed, and a clinical trial investigating the therapeutic benefits of the mediterranean diet on those living with PD (Medi-PD: NCT04683900)^114^ is ongoing. These early promising results warrant additional investigations of the gut microbiota in prodromal and early PD to assess effects on disease progression.

## Conclusion

The communication between the gut, the peripheral immune system, and the CNS is complex. Chronic systemic inflammatory (CSI) diseases, such as IBD, rheumatoid arthritis, and psoriasis, are epidemiologically associated with age-related neurodegenerative diseases, including PD. However, the evidence for a causal relationship between peripheral inflammatory processes and the development of neurodegeneration is still lacking and will be difficult to demonstrate in PD due to the slow development of disease. Directly consistent with the gut-first hypothesis of PD pathogenesis, several studies support the role of gut bacterial metabolites and/or environmental toxins as one of the main inflammation-associated mitochondrial stressors resulting in the chronic activation of the peripheral immune system in the blood and the gut^90^. Within this review, we have provided evidence that iron mishandling clearly occurs at multiple system levels throughout the course of PD and may further fuel this CSI. We propose that ferritin, the primary iron storage protein, may be an early molecular indicator of inflammation and associated mitochondrial stress in the gut that in the future could possibly be used as one of likely many biomarkers to diagnose early stages of PD. However, the role gut-specific iron regulation plays in peripheral immune cell dysfunction and how that may be a risk factor for PD etiopathogenesis is still understudied. Of importance is the understanding that most gut dysbiosis is modifiable and often induced by environmental factors. Understanding how to therapeutically target gut dysbiosis to prevent pathogenic and systemic inflammatory communication through the gut-blood-brain axis represents a promising and tractable goal that could be critically effective to reduce, delay, or arrest development of PD worldwide.

## Data Availability

No datasets were generated or analyzed during the writing of this review.

## Abbreviations

Aβ: amyloid beta
ACD: anemia of chronic disease
CSF: cerebrospinal fluid
CSI: chronic systemic inflammation
DA: dopamine
DOPA: dihydroxyphenylalanine
ETC: electron transport chain
GI: gastrointestinal
H_2_S: hydrogen sulfide
IBD: inflammatory bowel disease
MRI: magnetic resonance imaging
PD: Parkinson’s disease
PET: positron emission tomography
ROS: reactive oxygen species
SCFAs: short-chain fatty acids
SN: substantia nigra
SWI: susceptibility-weighted imaging
TDP-43: TAR DNA-binding protein 43
TH: tyrosine hydroxylase
TSPO-PET: translocator protein-positron emission tomography
UPDRS: Unified Parkinson’s Disease Rating Scale
